# Hearing the Voice of Medical Students Worldwide

**DOI:** 10.1371/journal.pmed.0020099

**Published:** 2005-04-26

**Authors:** Brian A Palmer, Amanda Wong, Mohit Singla

## Abstract

The Student Forum, a new section of *PLoS Medicine,* is a space where medical students from across the world can exchange ideas about the critical issues affecting health and health care from their unique perspective

## A World of Contrasts

Medical students today enter a profession defined by stark contrasts. In developed countries, public health improvements have nearly doubled life expectancy in the last century, and sophisticated technology and innovative research hold the promise of longer and higher quality life. At the same time, life-threatening infectious diseases such as tuberculosis and malaria continue to affect billions of people, and a rapidly escalating HIV/AIDS pandemic now kills more than 8,300 people each day [1]. New drugs are being developed at a record pace, but only about 1% of drugs that reach the market are aimed at treating neglected diseases, such as kala-azar, Chagas' disease, and sleeping sickness, even though these account for over 10% of the global disease burden [2]. Both within and between countries, the gap between wealthy and poor has continued to grow, leading to widening disparities in health outcomes.

Despite these contrasts, physicians-in-training around the globe—in rich and poor countries—have a common goal: we seek the skills and knowledge to improve the health of individuals and populations as we devote ourselves to a career of service. The transformative quest to become a physician imbues the learner with insights from within and outside the profession of medicine. As medical students, we hold ourselves accountable to our patients, and we critically evaluate the state of our profession as we see it with new eyes.

This issue of *PLoS Medicine* heralds the start of the Student Forum section, a space where medical students from across the world can exchange ideas about the critical issues affecting health and health care from their unique perspective. It is appropriate, then, in this inaugural Student Forum, that we consider some of the key issues of information exchange in the context of global disease and medical education. How do we access information? How is that information shaped and influenced? How can we shape the content of the debate?

## Open Access to the Medical Literature: Critical for Our Future

As physicians-in-training, we appreciate that the medicine we will practice will differ from that practiced by our predecessors. Mastery of access to information now trumps mastery of facts, and we enter an era of medical practice that will develop synergistically with biomedical research. Our generation of physicians will face the daunting task of translating an exploding body of biomedical and clinical research into medical practice. This task increasingly relies not only on the ability to access all the available medical literature, but on the ability to mine and synthesize it. PLoS represents one of a number of possible approaches toward ensuring free and open access to the world's literature [3], and students should be at the forefront of this movement.

As the democratization of access brings developed and developing countries together, the very content of the research published should follow suit. Too often the thoughts and opinions of those in the rich world have dominated research agendas and the content of medical journals. For example, less than 8% of articles published in the six leading tropical medicine journals in 2000–2002 were generated exclusively by scientists from developing countries [4]. Bringing the entire world of learners into a global conversation should be accompanied by a shift in editorial priorities to ensure that medical journals inform practice in a way that offers the greatest benefits to those suffering the greatest burdens.

**Figure:**
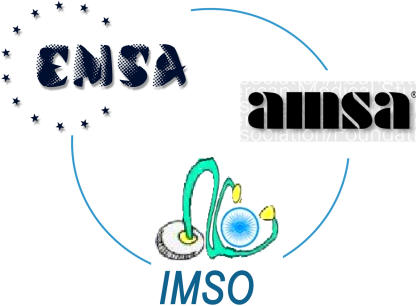
The Student Forum: A space where medical students across the globe can exchange ideas

## The Influence of the Pharmaceutical Industry

Journal editors and publishers pride themselves on publishing high-quality research subjected to rigorous peer review. Indeed, this model has contributed to significant advances in the standards for clinical investigation and rightly drives much of clinical practice. However, without the least bit of acknowledged irony, medical journals have begun publishing articles about how pharmaceutical industry advertising and marketing drives clinical practice in nonrational ways while at the same time publishing the very advertisements they critique.

The American Medical Student Association (AMSA), which has entered into a formal partnership with *PLoS Medicine*, accepts no pharmaceutical industry funding and encourages its members to eschew the industry largess through its “PharmFree” initiative (www.amsa.org/prof/pharmfree.cfm). Similar initiatives are afoot in medical student organizations around the world as we consider appropriate relationships with industry. Surely it is time that journals examine the content of their pages and stop serving as vehicles through which physician practice is influenced by industry. *PLoS Medicine* is the first major international general medical journal to refuse to advertise medical drugs or devices [5]. We hope that other major journals will follow suit.

## The Need for Student Ideals and Ideas

Throughout the world, students represent the leading edge of social change. Indeed Paulo Freire, one of the most influential educational thinkers of the late 20th century, describes education as the “practice of freedom,” as learners continuously work to integrate action and reflection in a spiral of deepening connections. This process of integration is rooted in a deep-seated longing for the possible, informed by the lived experience of the painful. Nowhere is this more true than in medicine, where the suffering of our patients underpins our scientific inquiry, our clinical development, and our personal growth as healers.

As we students learn medicine, we begin to ask hard questions about the proximal—and the distal—causes of our patients' suffering. The reductionistic logic of medical education offers only false comfort, and only to those who content themselves with pathways and receptors. Learners want to understand what led to the derangement of the pathway, of the receptor. Genes? Environmental factors? Economics? In asking, we bring a sense of possibility, of hope, to the process. What *could* be? What tools do we need to address these broader causes? And we hope that thinking about these questions from a student's view may offer our senior colleagues a chance to reflect on their own work as physicians and their own place in our world.

We hope this space emerges as a vibrant source of student energy and passion, grounded in evidence, accountable to the patients who will entrust their care to us, and reflecting the hope we have for the future of medicine.

## Toward the Future

As we come together from three continents to inaugurate the Student Forum in *PLoS Medicine*, we are committed to ensuring that this section of the journal remains a source of vigorous debate and thoughtful analysis. The section will initially be published quarterly, and will contain essays of up to 1,000 words that address any health-related issue from a medical student perspective (see Box 1).

**Box 1. Contributing to the Student Forum**

- *PLoS Medicine* welcomes essays of up to 1,000 words, which have not been published elsewhere, from medical students for the Student Forum.
- Before submitting an essay, please send a pre-submission enquiry to E-mail: studentforum@plos.org, explaining in 100 words what the essay will be about.
- The journal is also keen to forge alliances with medical student associations in Africa, Latin America, and Australia, and to elect a representative from each of these associations to join the *PLoS Medicine* student advisory group. Interested associations should contact the *PLoS Medicine* magazine editor (E-mail: gyamey@plos.org).

The *PLoS Medicine* medical student advisory group, which currently has representatives from the American Medical Student Association, the Indian Medical Students' Organization, and the European Medical Students' Association, will be involved in selecting and editing essays for publication.

The Student Forum will be freely available for all to read and critique, free from inappropriate financial interests, and committed to the drive toward justice that student ideals embody. We look forward to helping shape this forum in the best traditions of free exchange, and we look forward to collaboration with our senior colleagues to hold us to these standards.
